# Cardiac Deletion of Smyd2 Is Dispensable for Mouse Heart Development

**DOI:** 10.1371/journal.pone.0009748

**Published:** 2010-03-17

**Authors:** Florian Diehl, Mark A. Brown, Machteld J. van Amerongen, Tatyana Novoyatleva, Astrid Wietelmann, June Harriss, Fulvia Ferrazzi, Thomas Böttger, Richard P. Harvey, Philip W. Tucker, Felix B. Engel

**Affiliations:** 1 Department of Cardiac Development and Remodeling, Max-Planck-Institute for Heart and Lung Research, Bad Nauheim, Hessen, Germany; 2 Section of Molecular Genetics and Microbiology and Institute for Cellular and Molecular Biology, University of Texas at Austin, Austin, Texas, United States of America; 3 Developmental Biology Division, Victor Chang Cardiac Research Institute, Darlinghurst, New South Wales, Australia; 4 Dipartimento di Informatica e Sistemistica, Università degli Studi di Pavia, Pavia, Lombardia, Italia; Cincinnati Children's Hospital Medical Center, United States of America

## Abstract

Chromatin modifying enzymes play a critical role in cardiac differentiation. Previously, it has been shown that the targeted deletion of the histone methyltransferase, Smyd1, the founding member of the SET and MYND domain containing (Smyd) family, interferes with cardiomyocyte maturation and proper formation of the right heart ventricle. The highly related paralogue, Smyd2 is a histone 3 lysine 4- and lysine 36-specific methyltransferase expressed in heart and brain. Here, we report that Smyd2 is differentially expressed during cardiac development with highest expression in the neonatal heart. To elucidate the functional role of Smyd2 in the heart, we generated conditional knockout (cKO) mice harboring a cardiomyocyte-specific deletion of Smyd2 and performed histological, functional and molecular analyses. Unexpectedly, cardiac deletion of Smyd2 was dispensable for proper morphological and functional development of the murine heart and had no effect on global histone 3 lysine 4 or 36 methylation. However, we provide evidence for a potential role of Smyd2 in the transcriptional regulation of genes associated with translation and reveal that Smyd2, similar to Smyd3, interacts with RNA Polymerase II as well as to the RNA helicase, HELZ.

## Introduction

The formation of the heart is one of the most complex processes during vertebrate development being dependent on the orchestrated interplay of a variety of cell types and the precise intracellular regulation of transcriptional networks [1]–[2]. The complexity of its development renders the heart vulnerable to congenital diseases, affecting 1–2% of all newborns and being the leading cause of death in infants under 1 year [3]. Although, in recent years considerable progress has been made in defining the molecular mechanisms that control cardiac growth and differentiation at transcriptional level, far less is known about the epigenetic control of heart development imparted by chromatin remodeling enzymes. It has become increasingly evident that, in addition to the well-established roles of histone acetyltransferases (HATs) and histone deacetylases (HDACs) in cardiac differentiation, histone methyltransferases and demethylases are also essential in both cardiac [4]–[6] and skeletal [7]–[10] muscle development.

Functionally, methylation of lysine or arginine residues on histone tails, similar to a plethora of other post-translational histone modifications (e.g., phosphorylation, acetylation, SUMOylation, ubiquitylation), has been shown to recruit protein complexes affecting target gene expression at the transcriptional level [11]–[12]. This complexity in histone modifications might not only be seen as a simple code, but rather as an ingenious chromatin ‘language’ where different biological outcomes are defined by the combinatorial modification of basic building blocks [13]. Additionally, in contrast to histone acetylation, lysine residues can either be mono-, di- or tri-methylated, thereby adding an additional level of ‘histone code’ complexity. Interfering with the controlled action of histone methyltransferases by either loss of function or gain of function experiments therefore often results in a deleterious biological outcome due to disturbed proliferation and/or differentiation. This phenomenon is not only true for the heart [14], but can also be observed in a wide range of other organs and cell types [15]–[18].

Members of the SET and MYND domain containing (Smyd) family of proteins possess SET-dependent methyltransferase capacity and have been shown to be involved in the transcriptional control of cell differentiation and cell proliferation [19]–[21]. However, with the exception of Smyd1, little is known about the distinct functional relevance of Smyd family proteins during vertebrate development. Evidence for a critical role of Smyd proteins during organ development was first shown by the constitutive knockout of *Smyd1/m-Bop*, resulting in early embryonic lethality due to disruption of cardiac differentiation and morphogenesis [14]. This finding made the Smyd proteins interesting candidates for the control of cardiac growth and differentiation. Subsequent reports have further indicated that Smyd -family members are, indeed, critical regulators of cardiac as well as skeletal muscle development [14], [21]–[29].

We identified Smyd2 as a histone 3 lysine 36 (H3K36) methyltransferase with highest expression in heart and brain [20]. Functionally, methylation on H3K36 is most often associated with actively transcribed genes [20], [30]–[31]. Our *in vitro* studies, however, revealed that Smyd2 acts as a transcriptional repressor when bound to HDAC1 and the Sin3 repression complex [20]. More recent findings suggest that Smyd2 is also capable of H3K4 methylation when bound to Hsp90α, showing that the full spectrum of Smyd2 impact on transcriptional regulation is still largely unknown [32]. Furthermore, it has been shown that Smyd2 acts on non-histone targets by inhibiting the functional activity of p53 via methylation of p53, lysine 370 [33]. Thus, several lines of evidence support a role for Smyd2 in the regulation of proliferation and in tumor progression [20], [33]–[36].

Despite being highly expressed in heart and brain, a specific functional relevance for Smyd2 in these organs has not yet been described. To that end, we have examined its spatiotemporal expression during vertebrate cardiac development and performed loss of function experiments. We report quite unexpectedly that, while Smyd2 is expressed nearly exclusively in cardiomyocytes in high abundance around birth, its cardiac-specific deletion has no major discernable impact on normal heart development.

## Materials and Methods

### Animals and cardiomyocyte isolation

This investigation conforms to the Guide for the Care and Use of Laboratory Animals published by the US National Institutes of Health (NIH Publication No. 85-23, revised 1996). Animal experiments were approved by the local committee for care and use of laboratory animals (Regierungspräsidium Darmstadt, Gen. Nr. B 2/202). Ventricular cardiomyocytes from fetal (E17), 3-days-old (P3), and adult (>10 weeks, 200–250 g) Sprague Dawley rats (Charles Rivers, or own breed) were isolated and cultured as described [37]–[38].

### Plasmids and constructs

Myc-tagged Smyd2 and Smyd3 have been described previously [20].

### Western Blot analysis

Cardiac ventricles were washed in ice-cold PBS, minced and then homogenized and lysed by repeated sonication in cell lysis buffer (Cell Signaling) containing 1 mM PMSF and 1x protease inhibitor cocktail (Roche) on ice. After additional 15 min incubation, samples were centrifuged at 17.000×g at 4°C for 10 min to remove cell debris. Whole cell extracts from cultured cells were made using the same lysis buffer. Nuclear/cytosolic fractioning was performed using NE-PER Kit (Pierce) according to the manufacturer's protocol. Total histone fractions were isolated from pooled (n = 6) neonatal (P3–5) *Smyd2^flox/flox^* or *Smyd2* conditional knockout (cKO) mouse hearts using the EpiQuick™ Total Histone Extraction Kit (Epigentek) according to the manufacturers protocol. Protein concentration was determined using DC Protein Assay (Bio-Rad). Equal amounts of proteins were resolved on 4–12% Bis-Tris Gels (Invitrogen) and blotted onto PROTRAN® nitrocellulose membranes (Whatman). Membranes were blocked with 5% non-fat dry milk in Tris-buffered saline (TBS) with 0.1% TWEEN-20 for 1 hour at RT and then incubated with primary antibodies overnight at 4°C under gentle agitation. Antigen-antibody complexes were visualized using horseradish peroxidase-conjugated secondary antibodies (Amersham) and SuperSignal® West Femto substrate (Thermo) on a VersaDoc imaging system (Bio-Rad). The following antibodies have been used: rabbit anti-Smyd2, 1∶500 (Abcam), mouse anti-PARP, 1∶500 (Transduction Laboratories), mouse anti-HSP70, 1∶500 (Transduction Laboratories), rabbit anti-Pan-Actin, 1∶2000 (Cell Signaling), mouse anti-GAPDH, 1∶4000 (SIGMA), mouse anti-p53, 1∶200 (SIGMA), rabbit anti-Troponin-I, 1∶250 (Santa Cruz), rabbit anti-H3K4me1, rabbit anti-H3K4me2, rabbit anti-H3K4me3, rabbit anti-H3K36me1, rabbit anti-H3K36me3 (Millipore), rabbit anti-H3K36me2, rabbit anti-H4K20me3, (Epigentek), rabbit anti-Histone H3, rabbit anti-Histone H4, (Bethyl Laboratories). All histone antibodies were diluted 1∶1000.

### Real-time qPCR and semi-quantitative RT-PCR

RNA from mouse tissue was isolated using TRIZOL (Invitrogen) according to standard protocols. RNA from cultured cells was isolated using RNeasy Kit (Qiagen) including on-column DNase digest according to the manufacturer's protocol. For cDNA synthesis the RNA from >10 fetal heart ventricles and ≥3 postnatal heart ventricles was pooled and subjected to reverse transcription using M-MLV reverse transcriptase (SIGMA). RNA for expression profiling in different mouse tissues was isolated from 3 male neonatal animals and pooled for cDNA synthesis. cDNA was used for real-time qPCR or semiquantitative RT-PCR respectively. Real-time qPCR was performed in triplicates using Absolute™ QPCR SYBR® Green Fluorescin Mix (Thermo SCIENTIFIC) and Bio-Rad iCYCLER iQ5 Real time PCR instrument. Relative gene expression was calculated on the basis of ΔCt values to *Gapdh* or *ß-actin* as housekeeping genes. All primer pairs used for real-time qPCR and RT-PCR respectively are summarized in the supplementary information (Table S1).

### Immunofluorescence staining

For cryosections P1 neonatal mouse hearts were isolated, washed in cold PBS and then embedded in POLYFREEZE™ tissue freezing media (Polysciences Inc.). Embedded hearts were frozen in methylbutane on liquid nitrogen, sectioned (transverse, 10 µM). For immunostaining cryosections or cultured cells were fixed for 15 min in 4% formaldehyde. Permeabelization was performed in PBS + 0.5% Triton X-100 for 10 min at RT and blocking in 5% goat serum/0.2% TWEEN-20/PBS for 1 h at RT. Primary antibodies were diluted in blocking solution and incubated at 4°C for overnight. The following antibodies have been used for immunostaining: rabbit anti-Smyd2, 1∶75 (Abcam), mouse anti-Tropomyosin, 1∶100 (SIGMA), mouse anti-Caveolin-3, 1∶200 (Transduction Laboratories).

### Histological analysis

The hearts from 5 day old neonatal or adult mice were dissected and soaked in ice-cold 30 mM KCl/PBS to induce diastolic arrest, washed and subsequently fixed in 4% paraformaldehyde, dehydrated, embedded in paraffin and sectioned at 6 µM. Sections were stained with hematoxylin and eosin or Masson's trichrome according to standard protocol and examined by light microscopy.

### Magnetic Resonance Imaging

MRI experiments were carried out on a 7.0 T Bruker Pharmascan, operating at 300.51 MHz for ^1^H and equipped with a 300 mT/m gradient system, using a custom-built circularly polarized birdcage resonator and the Early Access Package for self-gated cardiac Imaging (Intragate, Bruker, Ettlingen, Germany) [39]. The measurement is based on a gradient echo method; the imaging parameters are: echo time/repetition time = 44.4/6.3 ms; flip angle = 15°; field of view = 2.20×2.20 cm; slice thickness = 1.0 mm; matrix = 128×128; repetitions = 100. The imaging plane was localized using scout images showing the 2- and 4-chamber view of the heart, followed by acquisition in short axis view, orthogonal on the septum in both scouts. Multiple contiguous short-axis slices consisting of 6 to 7 slices were acquired for complete coverage of the left ventricle. MRI data were analyzed using Qmass digital imaging software (Medis, Leiden, Netherlands). All mice were measured under volatile isoflurane (1.5 to 1.7%) anesthesia and body temperature was maintained at 37°C throughout the measurements. For measurement 4 male *Smyd2^fl/fl^* animals were used as control group compared to 4 male *Smyd2* cKO animals, all 6 months old.

### Cloning of the *Smyd2* conditional targeting construct and generation of *Smyd2* conditional knockout mice

To construct the *Smyd2* conditional targeting construct, two genomic fragments were first subcloned from the C57BL/6 murine Bac clone-RPC124288J3. A 2.2 kb KpnI fragment containing exon 1 and a KpnI fragment containing 5.2 kb of intronic sequence between exons 1 and 2 was subcloned into pBluescript (Stratagene). Fragment 1 (5.2 kb) was excised with KpnI, blunt ended, and ligated into the unique blunt ended SalI site of pDELBOY [40]. The resulting clones were screened for correct orientation and for the regeneration of the SalI site. Fragment 2 (2.2 kb) was excised with KpnI and ligated into the unique KpnI site of pDELBOY containing fragment 1. This was subsequently screened for correct orientation. Fragment 3, containing 0.6 kb upstream of exon 1, was generated using Platinum *Pfx* DNA Polymerase (Invitrogen), C57BL/6 genomic DNA as template, and the following primer pair 5′gtcgacattgagctaatgtgctta-3′and 5′-ctcgaggtaacactcaacctctgc-3′. The resulting PCR product was treated with Taq Polymerase, ligated into pGEM-T EASY (Promega), and excised with SalI and XhoI. This product was ligated into the unique XhoI site of pDELBOY containing fragments 1 and 2 and subsequently screened for correct orientation. The completed targeting construct was linearized at the short arm of homology using XhoI. C57BL/6 ES cells were then transfected and selected with G418 and gancyclovir. Targeted ES cell colonies were screened by Southern hybridization analysis using probes specific for the genomic sequence external to the arms of homology. The 5′ Southern used a 0.8 kb PCR fragment using the following primer pair: 5′-ggctggagttagaggtggttatga-3′and 5′-acagctctgggctcggaaataaag-3′. The 3′ Southern used a 0.9 kb PCR fragment using the following primer pair: 5′-aactccatgtggtggaattctgtggt-3′and 5′-gcagcctgaaagaatcccttagact-3′. Successfully targeted ES cells were identified by Southern analyses. These assays were performed across the long arm of homology and the short arm of homology of clones that had experienced homologous recombination at the *Smyd2* locus. A size difference allowed the separation of targeted allele and wild type allele. These clones were then injected into C57Bl/6J-tyr©-2J (an albino strain) blastocysts, which were implanted into the uteri of psuedopregant recipients and ultimately chimeras were born. The chimeras were mated to albino C57Bl/6 females and resulting progeny with a black coat color were genotyped. Mice that were shown to be heterozygous for the targeted allele were mated to Flip recombinase-expressing transgenic mice to remove the neo cassette. Targeted deletion of *Smyd2* in cardiac tissue was accomplished by crossing mice expressing Cre recombinase under the control of the *Nkx-2.5* promoter as described previously [41] into *Smyd2* homozygous floxed mice and backcrossing the resulting heterozygous mice governing the Cre recombinase back to homozygous *Smyd2^flox/flox^* mice. Cre mediated recombination resulted in a deletion of a region including exon 1 of *Smyd2*.

### Microarray analysis

RNA was isolated from postnatal day 5 (P5) *Smyd2^flox/flox^* or *Smyd2^flox/flox^Cre* mouse cardiac ventricles (n = 4/4) according to standard TRIZOL protocol (Invitrogen). RNA quality was assessed using Agilent 2100 Bioanalyzer and RNA 6000 Nano Kit (Agilent). For mRNA expression analysis, the Affymetrix GeneChip Mouse Gene 1.0 ST Array was employed with the Affymetrix total RNA labeling protocol. Data were analyzed by the RMA algorithm using the Affymetrix Expression Console. Annotation and statistical analysis were performed with the DNAStar™ Arraystar™ 3.0 software using log2 transformed data. Fold changes were calculated on the basis of the median of signal intensity of the groups. To maximize the number of true positives, unpaired t-test without further correction was used for statistical analysis. Microarray data is deposited in MIAME compliant format at the ArrayExpress Database (http://www.ebi.ac.uk/microarray) with the assigned accession number: E-MEXP-2542.

### Statistical analysis

Results were analysed by GraphPad Prism (version 4.00, GraphPad Software Inc.). Statistical significance was determined using a Student's *t*-test. Values of *P*<0.05 were considered statistically significant.

## Results

### Temporal expression profiling of Smyd-family members during cardiac development

**Figure 1:**
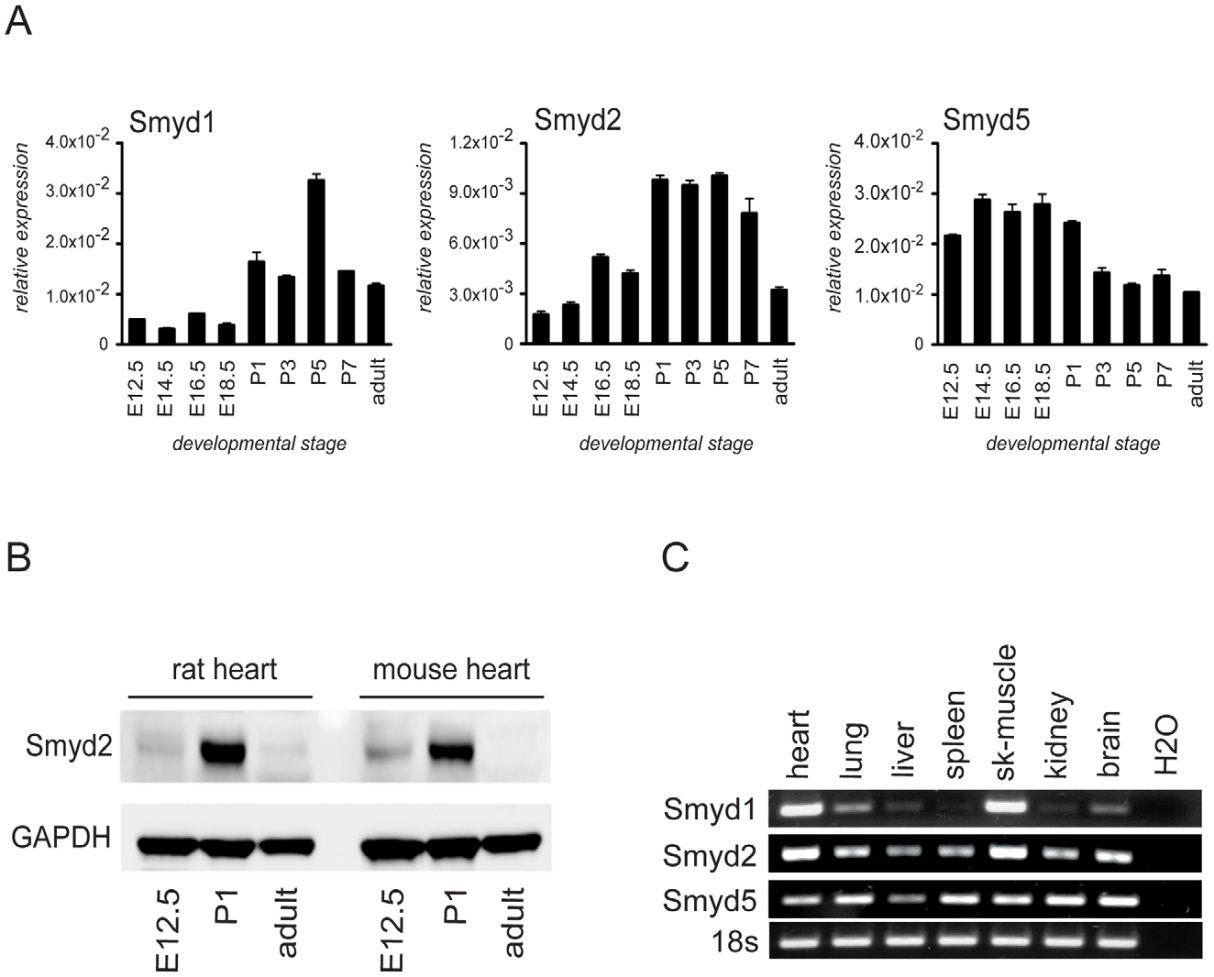
Temporal expression patterns of Smyd-family members during vertebrate heart development. (A) Real-time qPCR showing relative expression levels of Smyd-family members in mouse heart ventricles at different developmental stages (E12.5 to adult as indicated). Expression levels of Smyd-family members were normalized to GAPDH Ct values as housekeeping gene. qPCR analysis of Smyd-family members −1, −2 and −5 reveals a peak mRNA expression for Smyd1 and Smyd2 between postnatal day 1 to day 5 while Smyd5 mRNA shows highest expression levels during embryonic stages E14.5 to E18.5 ceasing after birth. (B) Western-blots of pooled E12.5, P1 or adult tissue extracts (70 µg) from rat and mouse heart ventricles were probed with anti-Smyd2 antibody showing maximal expression at postnatal day 1. Blots were re-probed with anti-GAPDH antibody for equal loading control. (C) Semiquantitative RT-PCR for Smyd1, −2 and −5 expression in neonatal (P4) mouse tissues. 18s rRNA expression was used as loading control.

To determine the temporal expression patterns of Smyd-family members during vertebrate heart development, we collected mouse cardiac ventricles at sequential developmental stages from embryonic (E) days 12.5 to 18.5, postnatal (P) days 1 to 7 and adult and determined the relative mRNA expression levels of Smyd1-5. Smyd1 and Smyd2 showed distinct expression in cardiac ventricles with peak mRNA expression between P1 and P5 displaying a >10-fold and >5-fold developmental change respectively. In contrast, Smyd5 expression changed less than 3-fold and peaked before birth (Fig. 1A). Smyd3 and Smyd4 expression was almost undetectable at any given time point (data not shown).

Since Smyd2 expression has not yet been described in the neonatal heart and previous expression analyses were only performed at the mRNA level using Northern-blotting and *in situ* hybridization [20], we next investigated Smyd2 protein expression in heart ventricles during cardiac development. Western-Blot analysis showed relatively high Smyd2 protein expression in P1 cardiac ventricles while very low expression was detected at E12.5 and adult. This expression pattern was conserved between rat and mouse (Fig. 1B).

As Smyd2 expression in the heart peaks shortly after birth, we re-evaluated Smyd2 expression in other organs at that time as our previous evaluation was performed only in adult mouse organs where Smyd2 expression is almost absent [20]. In line with our previous findings, Smyd2 showed a broader organ distribution pattern compared to Smyd1 but was most highly expressed in heart, skeletal muscle and brain tissue. In contrast, Smyd5 expression levels were uniform in all analyzed organs (Fig. 1C).

### Smyd2 is specifically expressed in cardiomyocytes

**Figure 2:**
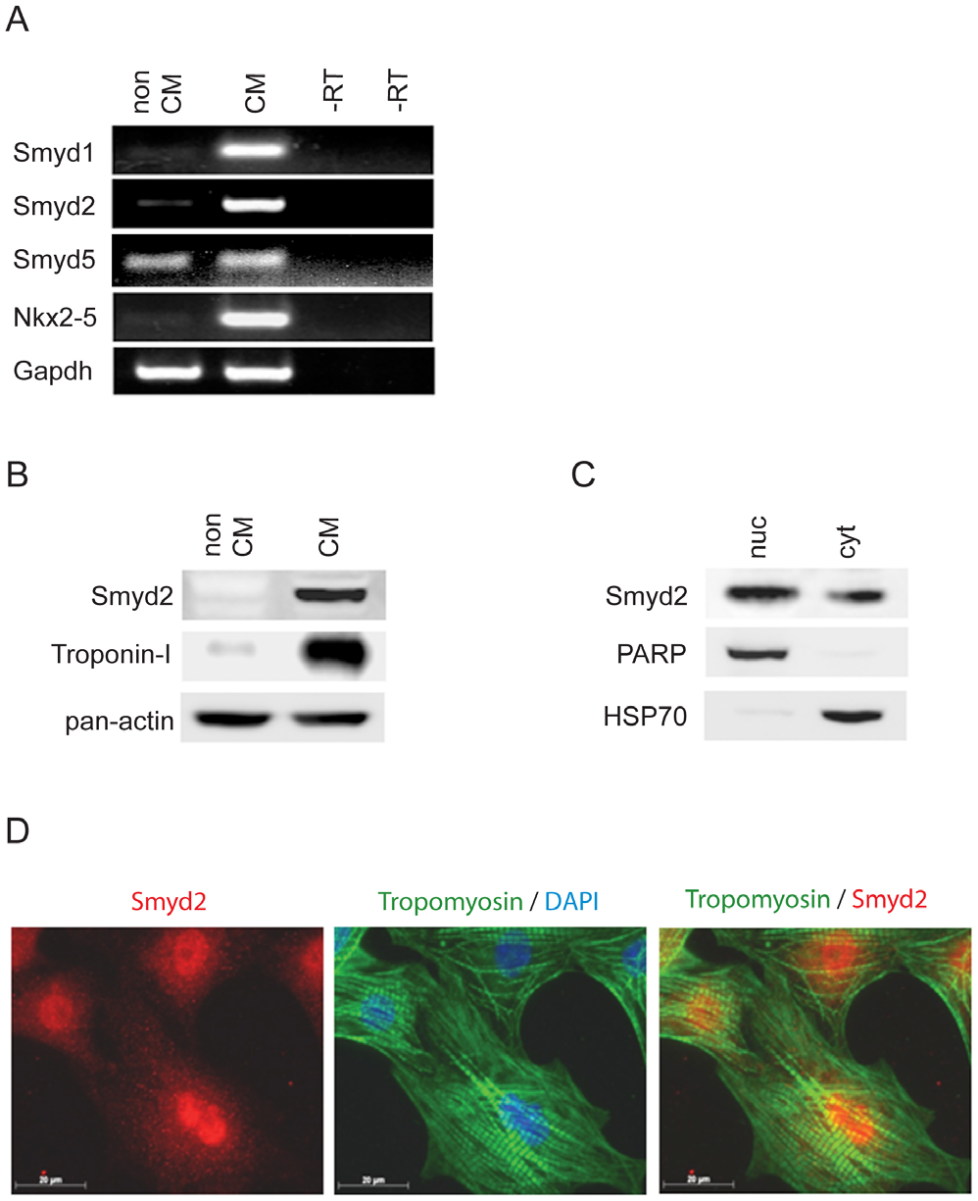
Smyd2 is predominantly expressed in cardiomyocytes. (A–B) RNA and protein was isolated from either non-cardiomyocyte (non-CM) or cardiomyocyte (CM) fraction following digestion of P3 rat heart ventricles. (A) Semi-quantitative RT-PCR was performed using primer pairs specifically detecting Smyd1, −2 and −5. Using RNA as a template served as negative control (-RT control). Smyd1 and Smyd2 show distinct expression predominantly in the cardiomyocyte fraction while Smyd5 is expressed in both fractions. The expression of the cardiac marker gene Nkx.2–5 was analyzed as a fraction purity control, GAPDH is shown as equal loading control. (B) Protein (50 µg) from non-cardiomyocyte and cardiomyocyte fractions was subjected to Western-blotting and blots were probed with anti-Smyd2 antibody showing a predominant expression of Smyd2 in the cardiomyocyte fraction. The membrane was re-probed with an antibody against cardiac Troponin-I as a control for fraction purity as well as anti-pan-actin antibody for controlling equal loading. (C) Nuclear and cytosolic fractions were assessed from P3 rat cardiomyocytes and equal protein amounts (50 µg) were subjected to Western-blotting. Smyd2 was detectable in nuclear as well as cytosolic fractions using anti-Smyd2 antibody. Blots were re-probed with antibodies against Poly (ADP-Ribose) Polymerase (PARP) as a nuclear marker protein or Heat shock protein 70 (Hsp70) as a cytosolic marker protein to assure fraction purity. (D) Immunocytochemistry using an anti-Smyd2 antibody shows that Smyd2 is expressed in the nuclei as well as in the cytoplasm of cultured P3 rat cardiomyocytes (red). Cardiomyocytes were co-stained using an antibody against cardiac Tropomyosin (green), nuclei were stained with DAPI (blue).

In order to obtain deeper insight into the cell type-specific expression characteristics of Smyd1, −2 and −5 in the heart, we isolated RNA from either non-cardiomyocyte (non-CM) or cardiomyocyte (CM) fraction from neonatal (P3) rat heart ventricles and performed semiquantitative RT-PCR. Fraction purity was determined using primer pairs that specifically amplify transcripts of the cardiac marker gene Nkx.2–5. Interestingly, Smyd1 and −2 were almost exclusively expressed in cardiomyocytes while, in accordance with its organ distribution, Smyd5 exhibited relatively uniform expression in CM and non-CM fractions (Fig. 2A). Smyd3 and −4 did not show significant expression in either fraction (data not shown).

The observed cardiomyocyte-specific expression of Smyd2 was further analyzed at the protein level. Western-blots confirmed the distinct Smyd2 protein expression in the Troponin-I positive cardiomyocytes (Fig. 2B).

Since Smyd proteins have been shown to modify both histone [20] and non-histone [33] targets, we next investigated the cellular localization of Smyd2 in cardiomyocytes. Western-blot analysis of nuclear and cytosolic extracts from neonatal (P3) rat cardiomyocytes revealed that Smyd2 protein is expressed in nuclear as well as cytosolic cell fractions. The specific fraction marker proteins PARP (nucleus) and HSP70 (cytosol) were used to confirm the purity of the fractions (Fig. 2C). In line with western blot results, this subcellular distribution was also observed using immunocytochemistry (Fig. 2D).

Taken together, the data indicate that Smyd1 and Smyd2 are the only Smyd-family members observed to be preferentially expressed in cardiomyocytes, and their levels are markedly regulated during cardiac development.

### Generation of mice containing floxed *Smyd2* alleles

**Figure 3:**
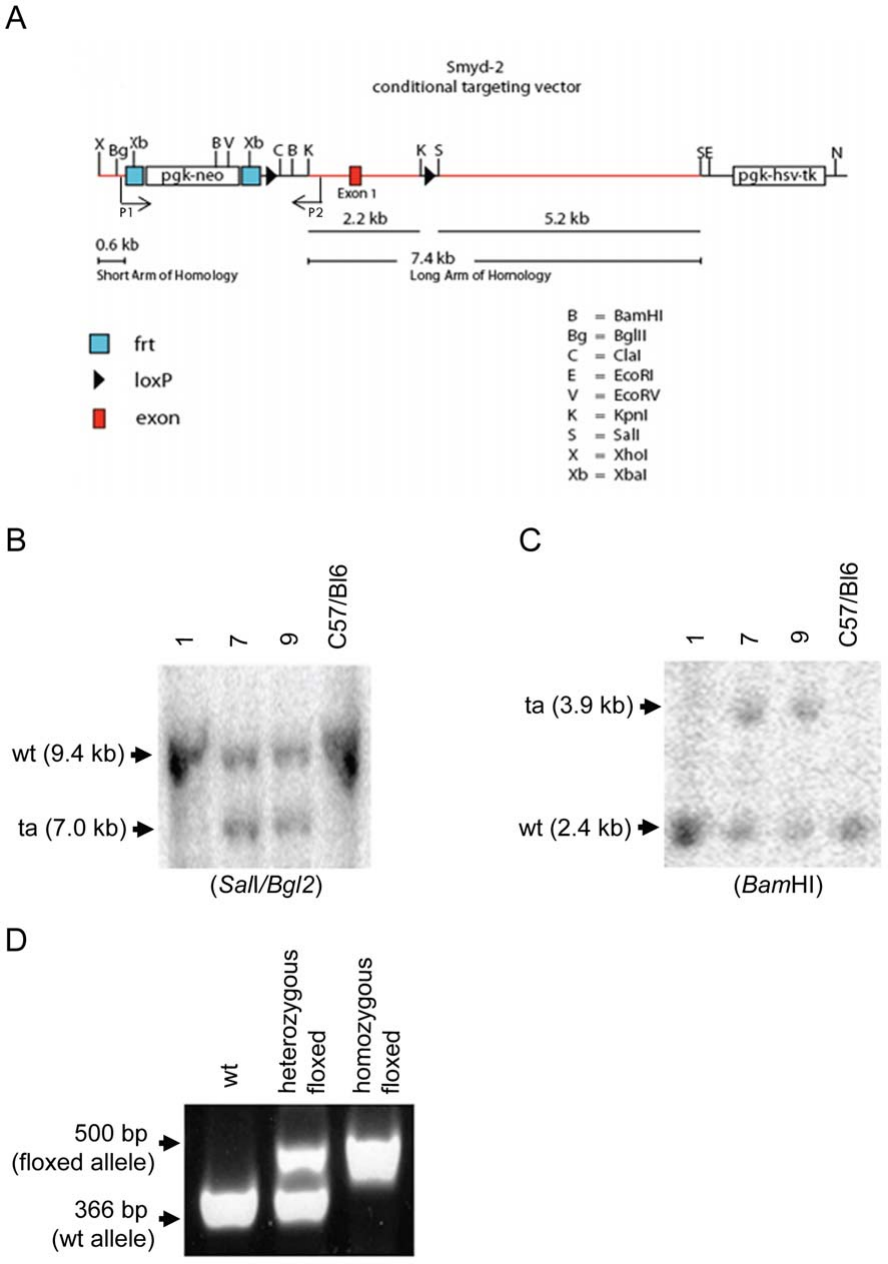
Generation of *Smyd2* conditional knockout mice. (A) The targeting vector contains a short (0.6 kb) and a long (7.4 kb) arm of homology, a neomycin resistance cassette (neo) for positive selection, and a thymidine kinase cassette for negative selection. Two site-specific recombination sites were employed *in vivo*. Flp recombinase was used to delete the neo marker from the mouse germline and Cre recombinase afterwards allowed the conditional deletion Smyd2 in selected tissues. Two loxP sites flank the region to be deleted. This region includes exon 1 of *Smyd2*. (B–C) Southern-blot analysis was performed to identify successfully targeted ES cell clones. Wild type (wt) or targeted (ta) alleles are indicated by arrows respectively. (B) Southern-blot analysis for homologous recombination was performed across the long arm of homology identifying clones 7 and 9 for successful recombination by size difference. (C) Southern-blot analysis for homologous recombination was performed across the short arm of homology also identifying clones 7 and 9 for successful recombination by size difference. The restriction enzymes used for Southern blotting were *Sal*I/*Bgl*2 or *Bam*HI as indicated below the blots. (D) Genotyping PCR was performed using a primer pair binding at positions P1 and P2 (A) to identify WT (366 bp) and floxed (500 bp) *Smyd2* alleles respectively. A representative genotyping PCR results for each genotype is shown (D).

Since Smyd2 expression is not restricted to the heart (Fig. 1C), we generated mice that allow the tissue specific deletion of *Smyd2* expression. For this purpose we generated *Smyd2* homozygous floxed mice (for detailed description see Materials and Methods). In brief, the targeting vector contained a short (0.6 kb) and a long (7.4 kb) arm of homology, a neomycin resistance cassette (neo) for positive selection, and a thymidine kinase cassette for negative selection. LoxP sites were positioned in a region including exon 1 of *Smyd2* (Fig. 3A).

Targeted ES cell colonies were screened by Southern hybridization analysis using probes specific for the genomic sequence external to the arms of homology. These assays were performed across the long arm of homology (Fig. 3B) using *Sal*I and Bgl2 for restriction and the short arm of homology (Fig. 3C) using *Bam*HI for restriction to select clones that had experienced homologous recombination at the *Smyd2* locus. Mice that were shown to be heterozygous for the targeted allele were mated to Flip recombinase-expressing transgenic mice to remove the neo cassette. Genotyping PCR was performed using a primer pair binding at positions P1 and P2 (Fig. 3A) to identify WT (366 bp) and floxed (500 bp) *Smyd2* alleles, respectively (Fig. 3D).

### Deletion of Smyd2 in cardiomyocytes

**Figure 4:**
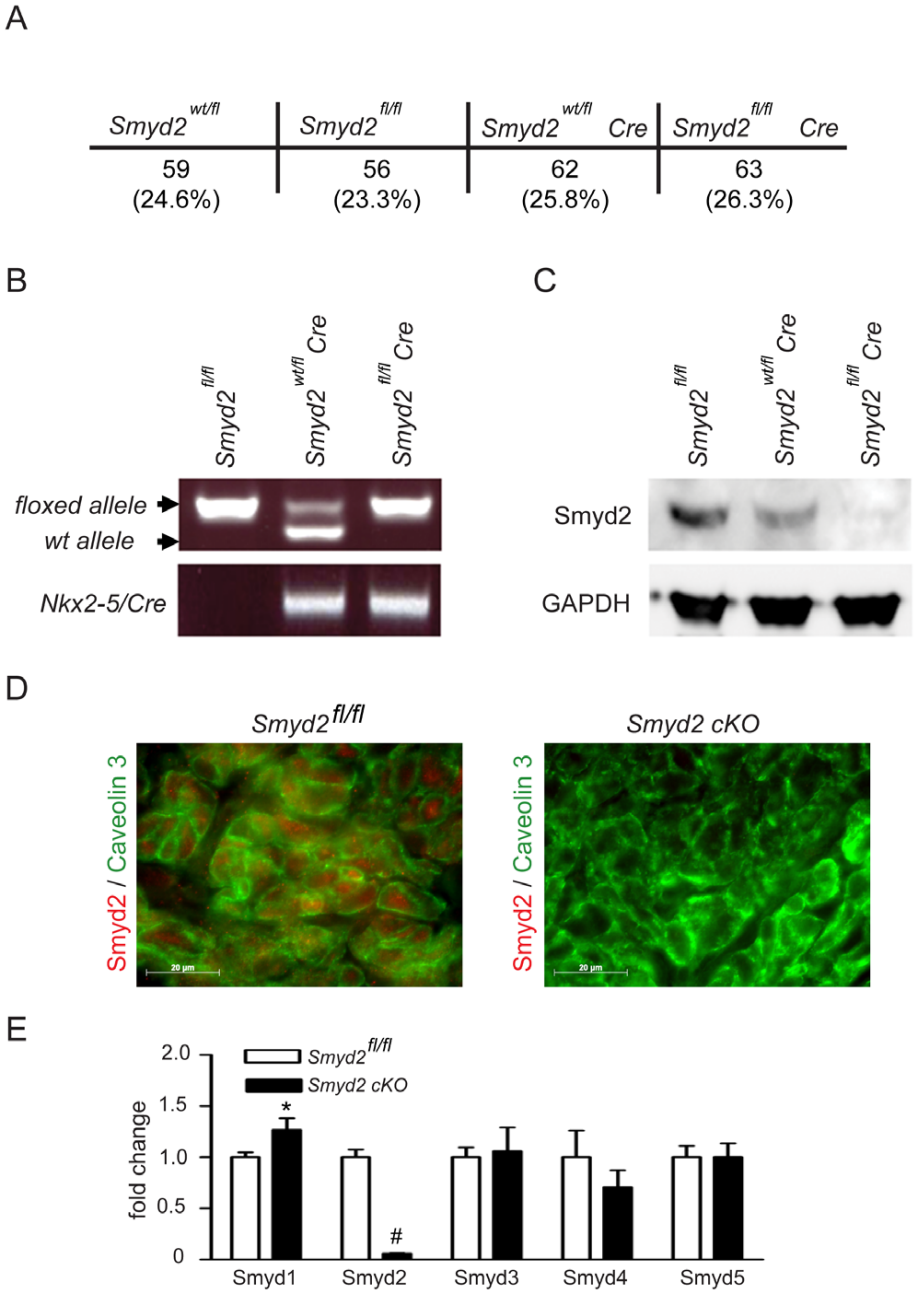
Analysis of cardiac specific Smyd2 deletion. (A) Mating of *Smyd2^fl/fl^* mice with *Smyd2^fl/fl^Cre* mice resulted in offspring of four genotypes (*Smyd2^wt/fl^, Smyd2^fl/fl^, Smyd2^wt/fl^Cre, Smyd2^fl/fl^Cre*) at normal Mendelian ratios. (B) The genotype of the animals used for analysis is shown by a representative genotyping PCR. Animals homozygous for the floxed *Smyd2* allele but lacking the *Nkx2–5* driven Cre recombinase were used as control. (C) Protein extracts (70 µg) from *Smyd2f^l/fl^*, *Smyd2^wt/fl^Cre* or *Smyd2^fl/fl^Cre* (cKO) mouse hearts were subjected to western-blot analysis and the knockdown efficiency as well as antibody specificity was assessed by probing the blot with an anti-Smyd2 antibody. Smyd2 protein expression was lowered by half in the heterozygous animals while it was almost completely absent in the homozygous cKO animals. The blot was re-probed with an anti-GAPDH antibody for equal loading control. (D) Cryosections from P1 control or *Smyd2* cKO mice were stained with an anti-Smyd2 antibody (red) and an anti-Caveolin-3 antibody (green) to co-stain the cardiomyocyte cell membrane. Smyd2 shows a distinct expression in the cardiomyocytes of control mice while no expression was observed in the cardiomyocytes of cKO animals. Pictures were taken at a magnification of 1000×. (E) Real-time qPCR showing relative expression levels of Smyd-family members 1–5 in P5 control or *Smyd2* cKO mouse heart ventricles. Data is shown as mean +/− SEM, *p<0.05 vs. control, #p<0.01 vs. control, n = 5.

Targeted deletion of Smyd2 in cardiomyocytes was accomplished by initially crossing mice expressing the Cre-recombinase under the control of the cardiac specific *Nkx2–5* promoter [41] with *Smyd2* homozygous floxed mice (*Smyd2^fl/fl^*). *Smyd2^fl/fl^* mice were mated with *Smyd2^wt/fl^* mice harboring the *Nkx2–5/Cre* transgene (*Smyd2^wt/fl^Cre*) to obtain *Smyd2* conditional knockout (cKO) mice (*Smyd2^fl/fl^Cre*). This mating resulted in offspring of 4 genotypes (*Smyd2^fl/fl^, Smyd2^wt/fl^*, *Smyd2^fl/fl^Cre, Smyd2^wt/fl^Cre*). As shown in figure 4A, animals were born at normal Mendelian ratios and survived until adulthood without any obvious abnormalities. For all subsequent analyses we used the *Smyd2^fl/fl^* genotype as the control group, as this is widely accepted [42]–[44]. The genotypes used for analysis are shown by a representative genotyping PCR (Fig. 4B). Western Blot analysis of cardiac tissue revealed that Smyd2 protein expression in *Smyd2^fl/wt^Cre* mice was approximately reduced by half, whereas Smyd2 expression was almost completely abolished in cKO (*Smyd2^fl/fl^Cre*) animals (Fig. 4C). Immunohistochemistry using cryosections from neonatal (P1) mouse hearts further confirmed loss of Smyd2 expression in cKO animals (Fig. 4D).

Since the loss of Smyd2 functional activity might result in compensation by other Smyd-family members, we also investigated whether expression levels of the related *Smyd* genes *1–5* are affected by Smyd2 deletion. Relative Smyd1–5 mRNA expression profiles in hearts from P5 mice were obtained using real-time qPCR of RNA/cDNA samples from either control or *Smyd2* cKO mice. This analysis confirmed the significant knockdown of Smyd2 expression and revealed that Smyd1/mBop expression was modestly, but statistically significantly, elevated (1.3-fold±0.09) in the cKO animals. No significant changes were detected for other Smyd transcripts (Fig. 4E).

### Morphologic and functional analysis of *Smyd2* cKO hearts

**Figure 5:**
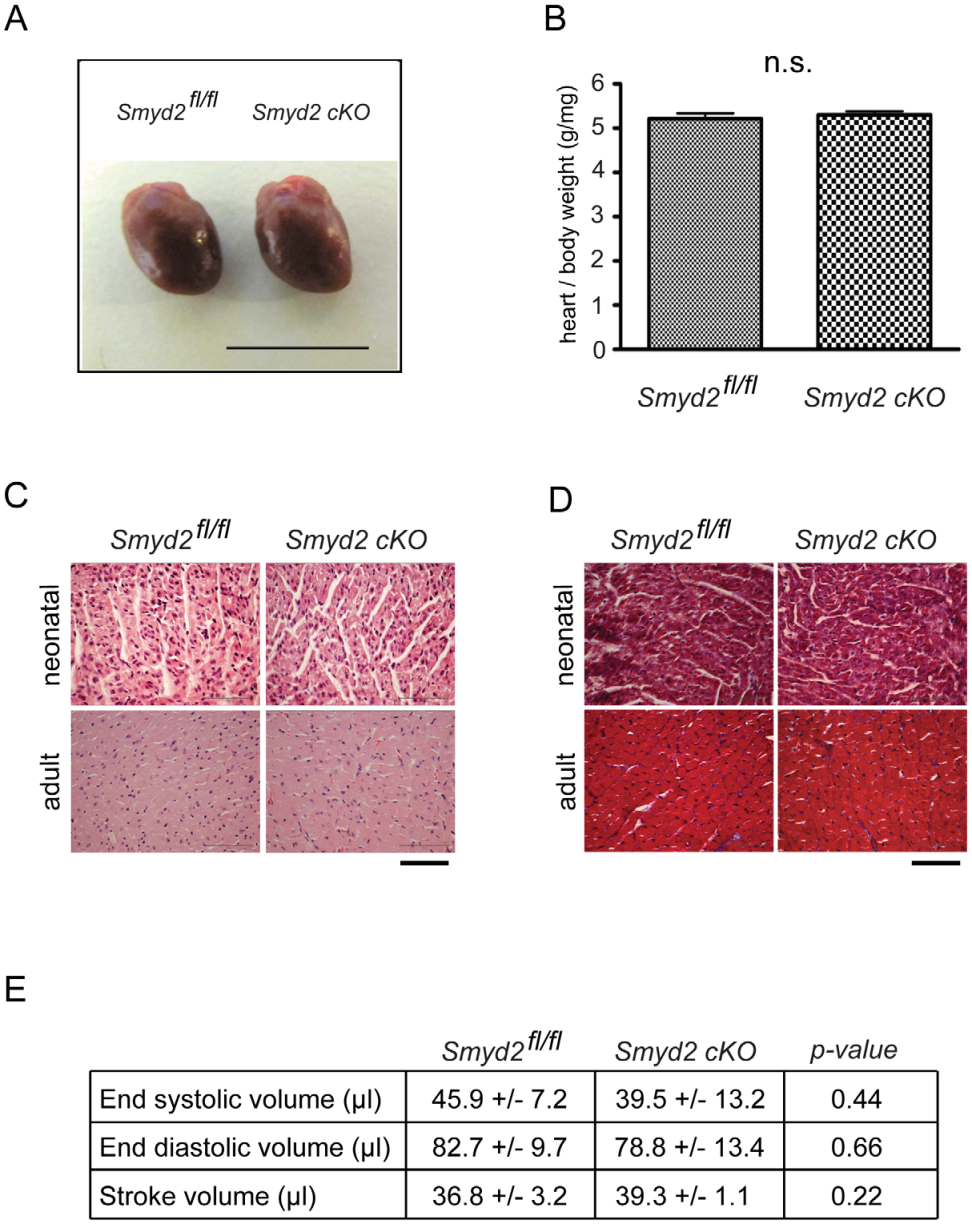
Morphologic analysis of *Smyd2* cKO hearts. (A) The hearts from 6 month old *Smyd2^fl/fl^* or *Smyd2* cKO animals were isolated and washed in cold 30 mM KCl/PBS. Subsequently photographs were taken using a standard digital camera. Scale bar indicates 1 cm. (B) Heart to body weight ratios were obtained from adult male *Smyd2^fl/fl^* or *Smyd2* cKO animals. Data is shown as means ± SEM, n = 4. (C) Tissue sections from neonatal (P3) or adult (>8 weeks) male *Smyd2^fl/fl^* or *Smyd2* cKO hearts were examined by histological analysis with hematoxylin and eosin staining (H&E). No signs of necrosis or cardiomyocyte disarray have been observed. Scale bar = 65 µm, n = 4. (D) Tissue sections from neonatal (P3) or adult (>8 weeks) male *Smyd2^fl/fl^* or *Smyd2* cKO hearts were examined by Masson's trichrome staining. Signs of fibrosis were not observed in cardiac ventricles. Scale bar = 65 µm, n = 4. (E) Functional cardiac parameters were obtained using MRI analysis of either male *Smyd2^fl/fl^* or *Smyd2* cKO mice at the age of 6 month. No statistical differences were detected for end-systolic, end-diastolic or stroke volume. Data is shown as means ± SD, n = 4.

Following successful knockdown of Smyd2 in cardiomyocytes we analyzed the hearts of adult *Smyd2* cKO mice. The *Smyd2* cKO hearts appeared normal (Fig. 5A), and we detected no significant changes in heart to body weight ratios compared to control mice at 6 month of age (Fig. 5B). Similar results were obtained from analyses of neonatal hearts (data not shown).

Since Smyd2 is most highly expressed after birth, we also examined the hearts of neonatal (P3) as well as adult (>8 weeks) mice by histological analysis using hematoxylin and eosin staining as well as Masson's trichrome staining. Upon microscopic observation we observed no obvious differences in necrosis or cardiomyocyte organization (Fig. 5C). Additionally, no signs of cardiac fibrosis were observed (Fig. 5D).

The absence of morphological changes would not rule out potential phenotypes at the functional level. Thus, we assessed functional cardiac parameters by MRI-analysis. As shown in Fig. 5E, Smyd2 deficiency did not alter end-systolic volume, end-diastolic volume or stroke volume compared to control littermates. Observed variations in functional parameters deviate in physiologic range [45].

Taken together, the data suggest that Smyd2 does not contribute non-redundantly to development or maintenance of normal cardiac morphology even though neonatal cardiomyocytes are the primary site of Smyd2 expression.

### Molecular analysis of *Smyd2* cKO hearts

**Figure 6:**
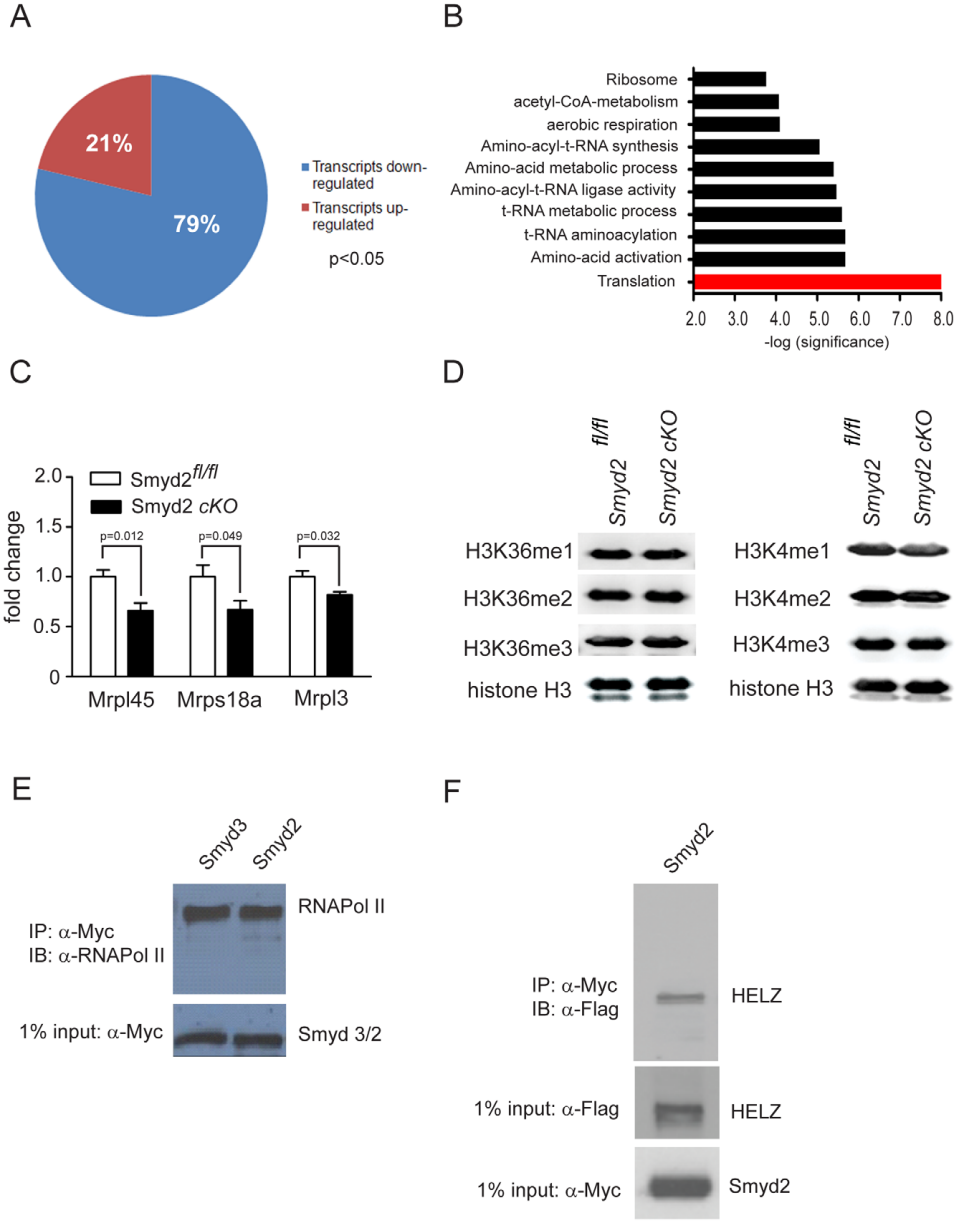
Molecular analysis of *Smyd2* cKO hearts. (A–B) Microarray analysis was performed on RNA from P5 neonatal *Smyd2^fl/fl^* or *Smyd2* cKO mouse heart ventricles (4 animals per genotype) and sorted for significantly regulated transcripts (p-value <0.05). (A) Significantly regulated transcripts have been sub grouped by either up- or down-regulation compared to the *Smyd2^fl/fl^* control group. (B) Gene ontology analysis was performed on significantly regulated transcripts using DAVID tool (http://david.abcc.ncifcrf.gov/). Significantly enriched biological processes are shown and plotted as the −log(p-value). (C) Real-time qPCR analysis of 3 nuclear encoded genes for mitochondrial ribosomal subunits was performed on RNA/cDNA from P5 neonatal *Smyd2^fl/fl^* or *Smyd2* cKO mouse heart ventricles. Data as shown as means ± SEM, n = 5. (D) Core histone fractions have been extracted from pooled neonatal (P3–P5) *Smyd2^fl/fl^* or *Smyd2* cKO hearts (6 hearts per genotype) and subjected to western blotting. Global histone methylation has been determined using antibodies against H3K36me1, −me2 and −me3 as well as H3K4me1, −me2 and −me3 as indicated. Pan histone H3 antibody was used for equal loading control. (E) Smyd2 interacts with RNA Polymerase II (RNAPolII). HeLa cells were transfected with either Smyd2-Myc, or Smyd3-Myc and 48 hours post-transfection whole RIPA lysates were used for immunopreciptation studies using an antibody directed against the Myc-epitope. Binding of RNAPolII to either Smyd2-myc or Smyd3-myc was subsequently determined by western-blot analysis using an RNAPolII-antibody. Input control for Smyd2-myc is shown in the lower panel. (F) Smyd2 associates with HELZ. HeLa cells were co-transfected with Smyd2-myc and HELZ-Flag plasmids and, 48 hours post-transfection, whole RIPA lysates were prepared. Antibodies directed against Myc were used for immunoprecipitation, followed by western analysis using antibodies directed against the Flag-epitope (upper panel). Input control is shown in lower panels.

In an attempt to reconcile these paradoxical findings, we investigated potential genome-wide changes in the cardiac transcriptome by expression profiling. Based on the observation that Smyd2 expression peaks after birth, we expected maximal changes in target gene expression between *Smyd2* cKO and control animals at that time. Yet, microarray analyses of neonatal (P5) heart ventricles revealed only modest changes in global gene expression patterns (ArrayExpress/accession no.: E-MEXP-2542). Although Smyd2 was previously shown to function as a transcriptional repressor in cultured cells [20], the vast majority (79%) of the differentially expressed transcripts were down-regulated (Fig. 6A). Functional annotation analysis for all significantly regulated transcripts [46]–[47] indicated that regulated transcripts are enriched in translational processes (p-value <0.01) (Fig. 6B). Interestingly, the majority of enriched transcripts in this cluster were found to encode for subunits of either cytosolic or mitochondrial ribosomes. Real-time qPCR was performed as a second technique to additionally confirm transcriptional regulation of certain mitochondrial ribosomal subunits (Fig. 6C).

The majority of deregulated genes in Smyd2 cKO hearts displayed reduced expression. This, along with the observation that Smyd2 gain of function experiments, *in vitro*, resulted in a predominant up-regulation rather than down-regulation of genes [32], predicted that Smyd2 might act as a transcriptional activator in the wild type heart. As Abu-Farah and colleagues found histone methyltransferase activity to be essential for target gene upregulation [32], we also determined the extent of H3K4 as well as H3K36 methylation in neonatal *Smyd2* cKO versus control hearts, as these histone modifications are generally believed to be associated with actively transcribed genes [30]–[31], [48]–[49]. However, western blot analyses using purified histone fractions revealed no differences in the extent of mono-, di- or tri-methylation on H3K36, or H3K4 (Fig. 6D).

An alternative mechanism by which Smyd2 might contribute to transcriptional activation was suggested in previous studies of the highly related paralogue, Smyd3 [19]. In addition to catalyzing H3K4 HMTase activity, Smyd3 may act as a direct transcriptional regulator via a ternary association with RNA Polymerase II and the RNA helicase, HELZ [19]. As with Smyd3, Smyd2 co-immunoprecipitates with co-transfected HELZ as well as with endogenous RNA polII (Fig. 6E, F). This feature, shared by Smyds2 and 3, might contribute to the observed target deregulation observed in our microarrays in the absence of global methylation changes.

## Discussion

Members of the Smyd protein family have been shown to be involved in the regulation of cellular differentiation processes [19]–[21]. It has become increasingly apparent that the functional role of Smyd proteins is of particular importance for the differentiation of muscle tissue [14], [25]–[27], [29]. Targeted gene disruption revealed Smyd1 to be essential for early cardiac development [14] by acting as a downstream effector of the cardiac transcription factor, MEF2C, in the developing heart [26]. However, functional characterization of other Smyd-family members in the heart has not been performed. Since we have recently identified Smyd2 as a distinct Smyd -family member that is most highly expressed in heart and brain [20], we performed a study aimed at expanding the understanding of Smyd proteins in the heart with specific focus on Smyd2.

Our results reveal that Smyd2 is differentially expressed during cardiac development, displaying highest expression levels around birth in rats and mice. In contrast to Smyd1 deficiency, loss of Smyd2 does not result in embryonic lethality, consistent with implications from expression data that Smyd2 functions later in development. *Smyd2* cKO animals are viable and are born in normal Mendelian ratios with no obvious changes in heart morphology or function. Thus, Smyd2 does not appear to be essential for early heart formation.

Given a peak expression of Smyd2 in the first week of postnatal life, one might anticipate that Smyd2 is important for the biological processes occurring during this time period, namely the irreversible exit from cell cycle [50]–[52] as well as the change from mainly lactate and glucose catabolism to mitochondrial fatty acid oxidation [53]. If Smyd2 was essential for these processes, we would have expected deregulation of genes associated with either cell cycle control (cyclins, CDKs, cell cycle inhibitors) or key regulatory enzymes for cardiac energy metabolism, such as *carnitine palmitoyl transferase-I* or *medium-chain acyl-CoA dehydrogenase* [54]. However, microarray analyses did not reveal significant changes of such marker genes in P5 mouse heart ventricles, nor did adult *Smyd2* cKO hearts exhibit differences in size or weight as would have been expected if the proliferation of cardiomyocytes was affected [55]. Surprisingly, we found that the majority of genes affected by cardiac Smyd2 deletion are functionally associated with translation. Interestingly, a number of the down regulated genes (eg, Mrpl45, Mrps18a and Mrpl3) belong to the nuclear encoded repertoire of mitochondrial ribosomal subunits [56]–[58]. To our knowledge there are no previous data showing a transcriptional increase in components of the translational machinery occurring after birth. Nonetheless, our results suggest that the hypertrophic growth of the heart just after birth might be facilitated by a temporary increase in protein translation. Such a phenomenon is consistent with previous results which demonstrated increased ribosome expression during pathologic hypertrophy of cardiomyocytes (for review see Hannan et al. [59]). The fact that we do not observe hypertrophy suggests that Smyd2 is not a key regulator of normal growth. It will, however, be of interest to test how *Smyd2* cKO mice react to stress.

We and others have previously characterized Smyd2 as a histone methyltransferase with capacity to methylate H3K36 [20] as well as H3K4 [32]. As these findings were based on *in vitro* as well as cell culture studies, our current study provided the opportunity to test whether corresponding effects could be observed *in vivo*. The observed absence of any detectable changes in global H3K36 or H3K4 methylation, while unexpected, indicates that redundant HMTases might compensate in the developing heart. In particular, Smyd1, also has H3K4 methyltransferase activity [25]. Since Smyd1 expression is slightly (but statistically significantly) elevated upon Smyd2 deletion, it is possible that this function of Smyd2 might be partially compensated by Smyd1. An alternative and trivial explanation might be that Smyd2 is predominantly expressed in cardiomyocytes which make up only 56% of all cell types in the murine heart [60]. Assuming that Smyd2 might be involved in the transcriptional regulation of a subset of target genes in cardiomyocytes, one might not expect to detect global changes in histone methylation using crude heart tissue by western blot techniques. Therefore, a more detailed analysis of histone methylation status on isolated murine cardiomyocytes at confirmed target site promoters will be conducted in future experiments.

The finding that most of the deregulated genes in Smyd2 cKO hearts were repressed indicate a role for Smyd2 as an activator in the developing heart. This is consistent with other data from overexpression studies, showing that Smyd2 gain of function predominantly results in the up-regulation of genes [32]. The finding that Smyd2 is capable of interacting with RNA Polymerase II as well as the RNA helicase, HELZ, suggests that Smyd2 might share functional similarities with Smyd3 [19]. Although we do not provide evidence for a functional consequence of the interaction between Smyd2 and RNAPolII or HELZ regarding the regulation of transcription, one might speculate that Smyd2 might also facilitate target gene expression via the elongation of transcription.

In addition to its molecular function as a histone methyltransferase, Huang et al. recently proposed a distinct role for Smyd2 as a putative oncogene by methylating p53 and thereby repressing its tumor suppressive function [33]. Although we did not specifically address the functional consequence of Smyd2 deficiency for p53 activity *in vivo*, one might have expected a pronounced phenotype, at least in adult Smyd2 cKO animals. This seemed reasonable, as it has been shown that cardiac deletion of Mdm4, another inhibitor of p53 functional activity, results in p53-dependent dilated cardiomyopathy [61]. However, functional misregulation of p53 by Smyd2 *in vivo* seems unlikely for the heart, as Smyd2 cKO hearts showed no noticeable change in the levels of apoptosis or necrosis, nor transcriptional changes in the p53 target genes Mdm2 and p21 (Figure S1A). Additionally we did not observe any differences in p53 protein stability (Figure S1B/C). Given the importance of understanding the precise mechanisms of p53 regulation *in vivo*, our Smyd2 cKO mice will provide a useful tool for gathering such information in the heart as well as other organs. The relevance of Smyd2 in the heart will be particularly interesting in regard to stress models (myocardial infarction, hypoxia), as functional misregulation of p53 and other stress sensors might be masked under physiologic conditions, becoming apparent only when an acute need is present.

In summary, our data reveal that Smyd2 is dispensable for cardiac development and maturation in the mouse under normal physiologic conditions. They further suggest that Smyd2 might be involved in the transcriptional regulation of genes associated with protein translation.

## Supporting Information

Table S1 (0.04 MB DOC)

Figure S1 Regulation of p53 target genes in Smyd2 cKO hearts (A) Microarray analysis was performed on RNA from P5 neonatal Smyd2fl/fl or Smyd2 cKO mouse heart ventricles. Transcriptional changes were analyzed for the p53 target genes Mdm2 and p21. Data is given as fold changes versus Smyd2flox/flox and shown as means ± SD, n = 4. (B) Protein extracts (50 µg) from P3 Smyd2fl/fl or Smyd2 cKO mouse hearts were subjected to western-blot analysis and blots were probed with an anti-p53 antibody. Blots were re-probed with an anti-GAPDH antibody for equal loading control. A representative blot is shown. No differences in p53 protein expression were observed. (C) Densitometric analysis of p53 protein expression using western-blot. Data is shown as means ± SEM and ratio to GAPDH, n = 3.
(0.54 MB TIF)

## References

[1] Srivastava D, Olson EN (2000). A genetic blueprint for cardiac development.. Nature.

[2] Rochais F, Mesbah K, Kelly RG (2009). Signaling pathways controlling second heart field development.. Circ Res.

[3] Nemer M (2008). Genetic insights into normal and abnormal heart development.. Cardiovasc Pathol.

[4] Bingham AJ, Ooi L, Kozera L, White E, Wood IC (2007). The repressor element 1-silencing transcription factor regulates heart-specific gene expression using multiple chromatin-modifying complexes.. Mol Cell Biol.

[5] Baskind HA, Na L, Ma Q, Patel MP, Geenen DL (2009). Functional conservation of asxl2, a murine homolog for the Drosophila enhancer of trithorax and polycomb group gene asx.. PLoS One.

[6] Shirai M, Osugi T, Koga H, Kaji Y, Takimoto E (2002). The Polycomb-group gene Rae28 sustains Nkx2.5/Csx expression and is essential for cardiac morphogenesis.. J Clin Invest.

[7] Caretti G, Di Padova M, Micales B, Lyons GE, Sartorelli V (2004). The Polycomb Ezh2 methyltransferase regulates muscle gene expression and skeletal muscle differentiation.. Genes Dev.

[8] McKinsey TA, Zhang CL, Olson EN (2002). Signaling chromatin to make muscle.. Curr Opin Cell Biol.

[9] Blais A, van Oevelen CJ, Margueron R, Acosta-Alvear D, Dynlacht BD (2007). Retinoblastoma tumor suppressor protein-dependent methylation of histone H3 lysine 27 is associated with irreversible cell cycle exit.. J Cell Biol.

[10] Rampalli S, Li L, Mak E, Ge K, Brand M (2007). p38 MAPK signaling regulates recruitment of Ash2L-containing methyltransferase complexes to specific genes during differentiation.. Nat Struct Mol Biol.

[11] Sims RJ, Nishioka K, Reinberg D (2003). Histone lysine methylation: a signature for chromatin function.. Trends Genet.

[12] Sims RJ, Reinberg D (2008). Is there a code embedded in proteins that is based on post-translational modifications?. Nat Rev Mol Cell Biol.

[13] Berger SL (2007). The complex language of chromatin regulation during transcription.. Nature.

[14] Gottlieb PD, Pierce SA, Sims RJ, Yamagishi H, Weihe EK (2002). Bop encodes a muscle-restricted protein containing MYND and SET domains and is essential for cardiac differentiation and morphogenesis.. Nat Genet.

[15] Madan V, Madan B, Brykczynska U, Zilbermann F, Hogeveen K (2009). Impaired function of primitive hematopoietic cells in mice lacking the Mixed-Lineage-Leukemia homolog MLL5.. Blood.

[16] Diehl F, Rossig L, Zeiher AM, Dimmeler S, Urbich C (2007). The histone methyltransferase MLL is an upstream regulator of endothelial-cell sprout formation.. Blood.

[17] Lubitz S, Glaser S, Schaft J, Stewart AF, Anastassiadis K (2007). Increased apoptosis and skewed differentiation in mouse embryonic stem cells lacking the histone methyltransferase Mll2.. Mol Biol Cell.

[18] Dodge JE, Kang YK, Beppu H, Lei H, Li E (2004). Histone H3-K9 methyltransferase ESET is essential for early development.. Mol Cell Biol.

[19] Hamamoto R, Furukawa Y, Morita M, Iimura Y, Silva FP (2004). SMYD3 encodes a histone methyltransferase involved in the proliferation of cancer cells.. Nat Cell Biol.

[20] Brown MA, Sims RJ, Gottlieb PD, Tucker PW (2006). Identification and characterization of Smyd2: a split SET/MYND domain-containing histone H3 lysine 36-specific methyltransferase that interacts with the Sin3 histone deacetylase complex.. Mol Cancer.

[21] Kwon C, Qian L, Cheng P, Nigam V, Arnold J (2009). A regulatory pathway involving Notch1/beta-catenin/Isl1 determines cardiac progenitor cell fate.. Nat Cell Biol.

[22] Peng YB, Yerle M, Liu B (2009). Mapping and expression analyses during porcine foetal muscle development of 12 genes involved in histone modifications.. Anim Genet.

[23] Kawamura S, Yoshigai E, Kuhara S, Tashiro K (2008). smyd1 and smyd2 are expressed in muscle tissue in Xenopus laevis.. Cytotechnology.

[24] Du SJ, Rotllant J, Tan X (2006). Muscle-specific expression of the smyd1 gene is controlled by its 5.3-kb promoter and 5′-flanking sequence in zebrafish embryos.. Dev Dyn.

[25] Tan X, Rotllant J, Li H, De Deyne P, Du SJ (2006). SmyD1, a histone methyltransferase, is required for myofibril organization and muscle contraction in zebrafish embryos.. Proc Natl Acad Sci U S A.

[26] Phan D, Rasmussen TL, Nakagawa O, McAnally J, Gottlieb PD (2005). BOP, a regulator of right ventricular heart development, is a direct transcriptional target of MEF2C in the developing heart.. Development.

[27] Thompson EC, Travers AA (2008). A Drosophila Smyd4 homologue is a muscle-specific transcriptional modulator involved in development.. PLoS One.

[28] Sims RJ, Weihe EK, Zhu L, O'Malley S, Harriss JV (2002). m-Bop, a repressor protein essential for cardiogenesis, interacts with skNAC, a heart- and muscle-specific transcription factor.. J Biol Chem.

[29] Li D, Niu Z, Yu W, Qian Y, Wang Q (2009). SMYD1, the myogenic activator, is a direct target of serum response factor and myogenin.. Nucleic Acids Res.

[30] Bannister AJ, Schneider R, Myers FA, Thorne AW, Crane-Robinson C (2005). Spatial distribution of di- and tri-methyl lysine 36 of histone H3 at active genes.. J Biol Chem.

[31] Rao B, Shibata Y, Strahl BD, Lieb JD (2005). Dimethylation of histone H3 at lysine 36 demarcates regulatory and nonregulatory chromatin genome-wide.. Mol Cell Biol.

[32] Abu-Farha M, Lambert JP, Al-Madhoun AS, Elisma F, Skerjanc IS (2008). The tale of two domains: proteomics and genomics analysis of SMYD2, a new histone methyltransferase.. Mol Cell Proteomics.

[33] Huang J, Perez-Burgos L, Placek BJ, Sengupta R, Richter M (2006). Repression of p53 activity by Smyd2-mediated methylation.. Nature.

[34] Scoumanne A, Chen X (2008). Protein methylation: a new mechanism of p53 tumor suppressor regulation.. Histol Histopathol.

[35] Sobral RA, Honda ST, Katayama ML, Brentani H, Brentani MM (2008). Tumor slices as a model to evaluate doxorubicin in vitro treatment and expression of trios of genes PRSS11, MTSS1, CLPTM1 and PRSS11, MTSS1, SMYD2 in canine mammary gland cancer.. Acta Vet Scand.

[36] Komatsu S, Imoto I, Tsuda H, Kozaki KI, Muramatsu T (2009). Overexpression of SMYD2 relates to tumor cell proliferation and malignant outcome of esophageal squamous cell carcinoma.. Carcinogenesis.

[37] Engel FB, Hauck L, Boehm M, Nabel EG, Dietz R (2003). p21(CIP1) Controls proliferating cell nuclear antigen level in adult cardiomyocytes.. Mol Cell Biol.

[38] Engel FB, Hauck L, Cardoso MC, Leonhardt H, Dietz R (1999). A mammalian myocardial cell-free system to study cell cycle reentry in terminally differentiated cardiomyocytes.. Circ Res.

[39] Larson AC, White RD, Laub G, McVeigh ER, Li D (2004). Self-gated cardiac cine MRI.. Magn Reson Med.

[40] Rossi DJ, Londesborough A, Korsisaari N, Pihlak A, Lehtonen E (2001). Inability to enter S phase and defective RNA polymerase II CTD phosphorylation in mice lacking Mat1.. EMBO J.

[41] Stanley EG, Biben C, Elefanty A, Barnett L, Koentgen F (2002). Efficient Cre-mediated deletion in cardiac progenitor cells conferred by a 3′UTR-ires-Cre allele of the homeobox gene Nkx2–5.. Int J Dev Biol.

[42] Iwasawa M, Miyazaki T, Nagase Y, Akiyama T, Kadono Y (2009). The antiapoptotic protein Bcl-xL negatively regulates the bone-resorbing activity of osteoclasts in mice.. J Clin Invest.

[43] da Costa Martins PA, Bourajjaj M, Gladka M, Kortland M, van Oort RJ (2008). Conditional dicer gene deletion in the postnatal myocardium provokes spontaneous cardiac remodeling.. Circulation.

[44] Pesu M, Watford WT, Wei L, Xu L, Fuss I (2008). T-cell-expressed proprotein convertase furin is essential for maintenance of peripheral immune tolerance.. Nature.

[45] Wiesmann F, Frydrychowicz A, Rautenberg J, Illinger R, Rommel E (2002). Analysis of right ventricular function in healthy mice and a murine model of heart failure by in vivo MRI.. Am J Physiol Heart Circ Physiol.

[46] Huang da W, Sherman BT, Lempicki RA (2009). Systematic and integrative analysis of large gene lists using DAVID bioinformatics resources.. Nat Protoc.

[47] Dennis G, Sherman BT, Hosack DA, Yang J, Gao W, Lane HC (2003). DAVID: Database for Annotation, Visualization, and Integrated Discovery.. Genome Biology.

[48] Lachner M, Jenuwein T (2002). The many faces of histone lysine methylation.. Curr Opin Cell Biol.

[49] Holbert MA, Marmorstein R (2005). Structure and activity of enzymes that remove histone modifications.. Curr Opin Struct Biol.

[50] Rumyantsev PP (1977). Interrelations of the proliferation and differentiation processes during cardiact myogenesis and regeneration.. Int Rev Cytol.

[51] Pasumarthi KB, Field LJ (2002). Cardiomyocyte cell cycle regulation.. Circ Res.

[52] van Amerongen MJ, Engel FB (2008). Features of cardiomyocyte proliferation and its potential for cardiac regeneration.. J Cell Mol Med.

[53] Lehman JJ, Kelly DP (2002). Transcriptional activation of energy metabolic switches in the developing and hypertrophied heart.. Clin Exp Pharmacol Physiol.

[54] Razeghi P, Young ME, Alcorn JL, Moravec CS, Frazier OH (2001). Metabolic gene expression in fetal and failing human heart.. Circulation.

[55] Hotta Y, Sasaki S, Konishi M, Kinoshita H, Kuwahara K (2008). Fgf16 is required for cardiomyocyte proliferation in the mouse embryonic heart.. Dev Dyn.

[56] Koc EC, Burkhart W, Blackburn K, Moyer MB, Schlatzer DM (2001). The large subunit of the mammalian mitochondrial ribosome. Analysis of the complement of ribosomal proteins present.. J Biol Chem.

[57] Kenmochi N, Suzuki T, Uechi T, Magoori M, Kuniba M (2001). The human mitochondrial ribosomal protein genes: mapping of 54 genes to the chromosomes and implications for human disorders.. Genomics.

[58] Ou JH, Yen TS, Wang YF, Kam WK, Rutter WJ (1987). Cloning and characterization of a human ribosomal protein gene with enhanced expression in fetal and neoplastic cells.. Nucleic Acids Res.

[59] Hannan RD, Jenkins A, Jenkins AK, Brandenburger Y (2003). Cardiac hypertrophy: a matter of translation.. Clin Exp Pharmacol Physiol.

[60] Banerjee I, Fuseler JW, Price RL, Borg TK, Baudino TA (2007). Determination of cell types and numbers during cardiac development in the neonatal and adult rat and mouse.. Am J Physiol Heart Circ Physiol.

[61] Xiong S, Van Pelt CS, Elizondo-Fraire AC, Fernandez-Garcia B, Lozano G (2007). Loss of Mdm4 results in p53-dependent dilated cardiomyopathy.. Circulation.

