# Supplementary material for: Cardiac Deletion of Smyd2 Is Dispensable for Mouse Heart Development
Source: PLoS One. 2010 Mar 17;5(3):e9748. doi: 10.1371/journal.pone.0009748 (PMC2840034; doi:10.1371/journal.pone.0009748)
Supplement: Table S1 — (0.04 MB DOC) [file pone.0009748.s001.doc]

**Table S1**

| **Target gene** | **forward primer (5’-3’)** | **Reverse primer (5’-3’)** |
| --- | --- | --- |
| **mouse *Smyd1*** | cctccactgtcgttttggtt | ccctctgcatagagctcacc |
| **mouse *Smyd2*** | ggagggccaaacactacaaa | tgagggagtacacggggtag |
| **mouse *Smyd3*** | tgatgaaagttggcaagctg | gtccttctgggggtccttg |
| **mouse *Smyd4*** | aggctgctgagagcttcttg | catggccaatttcaacactg |
| **mouse *Smyd5*** | gtcaccgacaccctccttta | aggcctctcagggaacaaat |
| **mouse *Mrpl45*** | ctaagcagcccatcctcaag | ccacaactgctaggctcctc |
| **mouse *Mrps18a*** | cacagaccacagcttccaga | tcagaagtcctgctcccact |
| **mouse *Mrpl3*** | ggactgccaccaaaacagat | tgtgggtctttgtttgacca |
| **mouse *Gapdh*** | aactttggcattgtggaagg | acacattgggggtaggaaca |
| **mouse *beta-Actin*** | tgttaccaactgggacgaca | ggggtgttgaaggtctcaaa |
| **rat *Smyd1*** | tgacagtaggcagcatggag | gtcaaaaaccactgccgaat |
| **rat *Smyd2*** | tgagggagtacacggggtag | gaccctggcagaagtcagag |
| **rat *Smyd3*** | aacgtcttcccgacatcaac | cagcttgccaactttcatca |
| **rat *Smyd4*** | actccaggaggcaagacaga | ctccaaggctttggtgagag |
| **rat *Smyd5*** | cactgggtcaggctcttctc | ccatctggtgtgaaccactg |
| **rat *Nkx2-5*** | cggtggagctggacaaagcc | tagcggcggttctggaacca |
| **rat *Gapdh*** | cagaagactgtggatggccc | agtgtagcccaggatgccct |
