# Supplementary figures and images for: Cardiac Deletion of Smyd2 Is Dispensable for Mouse Heart Development
Source: PLoS One. 2010 Mar 17;5(3):e9748. doi: 10.1371/journal.pone.0009748 (PMC2840034; doi:10.1371/journal.pone.0009748)

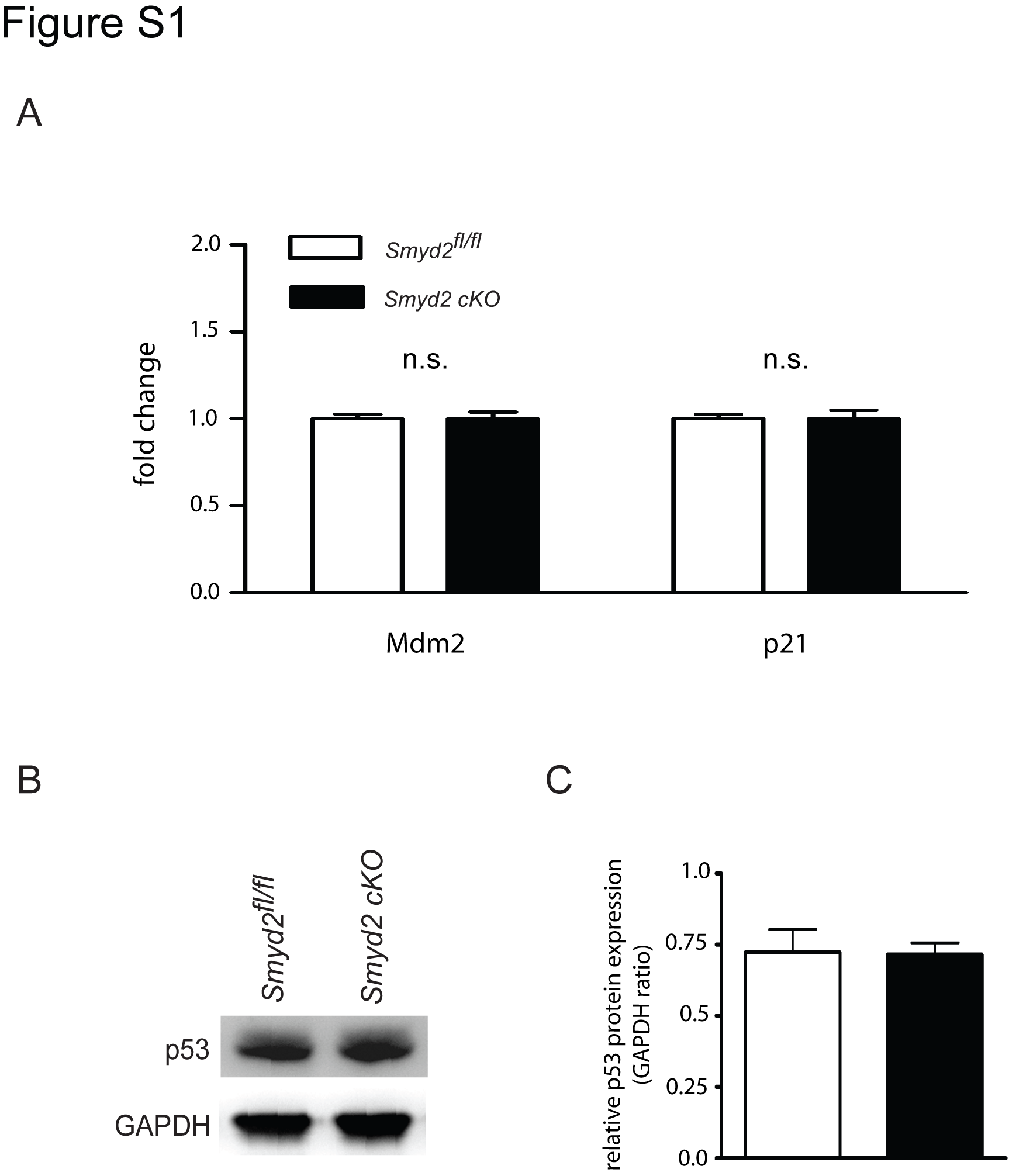

Supplement: Figure S1 — Regulation of p53 target genes in Smyd2 cKO hearts (A) Microarray analysis was performed on RNA from P5 neonatal Smyd2fl/fl or Smyd2 cKO mouse heart ventricles. Transcriptional changes were analyzed for the p53 target genes Mdm2 and p21. Data is given as fold changes versus Smyd2flox/flox and shown as means ± SD, n = 4. (B) Protein extracts (50 µg) from P3 Smyd2fl/fl or Smyd2 cKO mouse hearts were subjected to western-blot analysis and blots were probed with an anti-p53 antibody. Blots were re-probed with an anti-GAPDH antibody for equal loading control. A representative blot is shown. No differences in p53 protein expression were observed. (C) Densitometric analysis of p53 protein expression using western-blot. Data is shown as means ± SEM and ratio to GAPDH, n = 3. (0.54 MB TIF) [file pone.0009748.s002.tif]
